# Learning to Train and to Explain a Deep Survival Model with Large-Scale Ovarian Cancer Transcriptomic Data

**DOI:** 10.3390/biomedicines12122881

**Published:** 2024-12-18

**Authors:** Elena Spirina Menand, Manon De Vries-Brilland, Leslie Tessier, Jonathan Dauvé, Mario Campone, Véronique Verrièle, Nisrine Jrad, Jean-Marie Marion, Pierre Chauvet, Christophe Passot, Alain Morel

**Affiliations:** 1 Laboratoire Angevin de Recherche en Ingénierie des Systèmes (EA7315), Université d’Angers, 49035 Angers, France; 2 Unité de Génomique Fonctionnelle, Institut de Cancérologie de l’Ouest Nantes-Angers, 49055 Angers, France; 3 Département d’Oncologie Médicale, Institut de Cancérologie de l’Ouest Nantes-Angers, 49055 Angers, France; 4 Institut de Cancérologie de l’Ouest Nantes-Angers, 49055 Angers, France; 5 Univ Angers, Nantes Université, Inserm, CNRS, CRCI2NA, SFR ICAT, 49035 Angers, France; 6 Département d’Anatomie et de Cytologie Pathologiques, Institut de Cancérologie de l’Ouest Nantes-Angers, 49055 Angers, France

**Keywords:** TCGA, ovarian cancer, RNA-seq, survival analysis, deep learning, molecular pathways

## Abstract

**Background/Objectives:** Ovarian cancer is a complex disease with poor outcomes that affects women worldwide. The lack of successful therapeutic options for this malignancy has led to the need to identify novel biomarkers for patient stratification. Here, we aim to develop the outcome predictors based on the gene expression data as they may serve to identify categories of patients who are more likely to respond to certain therapies. **Methods:** We used The Cancer Genome Atlas (TCGA) ovarian cancer transcriptomic data from 372 patients and approximately 16,600 genes to train and evaluate the deep learning survival models. In addition, we collected an in-house validation dataset of 12 patients to assess the performance of the trained survival models for their direct use in clinical practice. Despite deceptive generalization capabilities, we demonstrated how our model can be interpreted to uncover biological processes associated with survival. We calculated the contributions of the input genes to the output of the best trained model and derived the corresponding molecular pathways. **Results:** These pathways allowed us to stratify the TCGA patients into high-risk and low-risk groups (*p*-value 0.025). We validated the stratification ability of the identified pathways on the in-house dataset consisting of 12 patients (*p*-value 0.229) and on the external clinical and molecular dataset consisting of 274 patients (*p*-value 0.006). **Conclusions:** The deep learning-based models for survival prediction with RNA-seq data could be used to detect and interpret the gene-sets associated with survival in ovarian cancer patients and open a new avenue for future research.

## 1. Introduction

Ovarian cancer is one of the most common female malignant tumor types and the fifth leading cause of cancer-related mortality in women worldwide [1]. Because it is usually diagnosed at a late stage and currently lacks effective treatment options, the five-year survival rate in advanced stage disease is as low as 30% [1]. The first-line therapy for ovarian cancer patients consists of cytoreductive surgery and platinum-based chemotherapy; although 80% of newly diagnosed patients respond to first-line therapy, approximately 75% with advanced stages experience disease relapse [2].

Currently, the additional major therapeutic regimen is a targeted Poly(ADP-ribose) polymerase (PARP) inhibitor, which can delay the progression of ovarian cancer. The clinicians have a choice of treatments that can be molecularly tailored to patients with breast cancer gene (*BRCA*) mutations, patients with homologous recombination deficiency (HRD), or patients regardless of their *BRCA* or HRD status [3]. Unfortunately, it is either significantly beneficial to the *BRCA*/HRD patients and/or hampered by the resistance phenomenon [4]. Therefore, there is an urgent need to identify and validate novel, highly sensitive, and specific molecular biomarkers for prognosis, monitoring, and therapy improvement in patients with HRD-negative disease or patients with resistance to PARP inhibitors.

The development of outcome predictors is important not only for patient stratification but also to identify categories of patients who are more likely to respond to certain therapies [5]. Several studies have attempted to develop molecular signatures based on gene expression to predict survival in ovarian cancer patients [6,7]. However, only a small number of prognostic signatures have been developed, and none have been directly applied in clinical practice [8].

Recent advances in neural networks initially led to the development of the survival models based on feed-forward networks [9,10,11,12,13,14,15]. The studies [7,16] extended the work of [9], which used the Cox negative partial likelihood as the loss function, to The Cancer Genome Atlas (TCGA) high-throughput transcriptomic data and developed SurvivalNet and Cox-nnet models, respectively. These deep learning models are designed to transform the high-dimensional gene expression inputs into more predictive lower-dimensional disease or biological features, but they can suffer from the so-called dimensionality curse and are prone to overfitting.

Variational Auto-Encoders (VAEs) [17] are another deep neural network architecture that generate latent representations for images and text. The work [18,19] explored the possibility of whether VAEs can be applied to gene expression data and whether they can capture biologically relevant features. The authors used the TCGA pan-cancer RNA-seq data to identify the patterns in the features learned by the VAE and discussed the potential benefits of this approach.

In order to improve the survival analysis using human cancer transcriptomic data and to overcome the overfitting problem, the VAEs were used by the authors of [20]. Their model VAECox first pre-trained a VAE network for dimensionality reduction on 20 transcriptomic TCGA datasets and fine-tuned it on 10 TCGA datasets using Cox negative partial likelihood for survival prediction. The authors reported that VAECox outperforms Cox-nnet [7] on 7 of the 10 TCGA datasets, including marginal improvement on the TCGA ovarian cancer (TCGA-OV) dataset in terms of concordance index.

Another deep learning approach used for data generation, reconstruction, and dimensionality reduction is the Generative Adversarial Networks (GANs) proposed by [21]. GANs adopt an adversarial training strategy, which effectively learns the distribution of high-dimensional data and effectively generates results sampled from the entire data distribution. The more recent Transformer architecture [22] with its attention mechanism has been widely used since its introduction. The attention function draws the global dependencies between the input and the output by mapping a query and a set of key–value pairs to an output.

The SAVAE-Cox model, recently proposed by [23], combines the adversarial transfer learning strategy and the attention mechanism. In the pre-training phase, the generator VAE is trained together with the discriminator, and the attention mechanism improves the extraction of semantically relevant features of the encoder from the high-dimensional data. After the pre-training, the generator learns the common features of 33 TCGA transcriptome datasets. Then, the encoder of the generator is trained for the survival analysis on 16 TCGA cancer types by minimizing the Cox negative partial likelihood.

Despite the integration of the recent advances in deep learning and the diversity of the TCGA datasets, the SAVAE-Cox model fails to improve the performance of Cox-nnet [7] on the TCGA-OV dataset. Furthermore, the performed ablation studies show that the proposed model without pre-training performs even better than with pre-training. In addition, the authors reported that VAECox [20] does not perform better than the state-of-the-art Cox-nnet on the TCGA-OV dataset. On the other hand, the paper [24] showed that training a neural network with the “pan-gyn” group of TCGA datasets [25] could improve the TCGA-OV prognostication. The use of other loss functions for survival analysis, such as in the N-MTLR model [12], improves the performance of the feed-forward neural networks on the TCGA-OV dataset compared to the Cox-nnet model [26].

Other common problems of the deep learning techniques are the overfitting and generalization failure. When applied to high-dimensional transcriptomic data, the deep learning survival models perform well on the training datasets and fail to generalize well to the test datasets or to transfer the learned features to the independent datasets. The studies of the models Cox-nnet [7], VAECox [20], SAVAE-Cox [23] evaluated the performance on the publicly available TCGA-OV dataset but did not use any external validation dataset to assess the generalization ability of the trained networks or to validate the significance of the detected molecular pathways.

The objective of this paper is to further explore the discrete-time survival model N-MTLR [12] by proposing its improved variant, which we call N-MTLR-Rank. We train this improved model using TCGA ovarian cancer clinical and molecular data. We use the Bayesian optimization technique [27] to automatically search the hyperparameter space, the different regularization techniques, and transfer learning by integrating the TCGA “pan-gyn” cohort [25] into the training phase. We seek to validate our deep learning survival model on an in-house clinical and molecular dataset. We investigate how the deep learning model can be interpreted by calculating the contributions of the input features to the network outputs. We demonstrate how these contributions can be related to the molecular pathways to uncover the biological processes associated with survival in ovarian cancer patients. We show that the found molecular pathways can indeed stratify the TCGA patients into high-risk and low-risk groups. Finally, we validate the stratification ability of the identified pathways on the in-house dataset as well as on the external clinical and molecular dataset. The significance of the obtained stratification provides further insights into the biological processes related to the survival of ovarian cancer patients and opens a new direction for future research.

The outline of the paper is as follows: Section 2 first describes the training and the validation datasets as well as the proposed discrete-time deep survival model. Then, it gives the technical details of the model training, validation, and interpretation stages. Section 3 presents the results of the training, validation, and interpretation, and Section 4 discusses the obtained results and future work.

## 2. Materials and Methods

### 2.1. Data

#### 2.1.1. TCGA Gene Expression and Clinical Data

We used the data obtained from our previous work as described in [24,26]. TCGA RNA-sequencing and clinical data were downloaded from the Genomics Data Commons (GDC) portal using the pipeline of the R/Bioconductor package TCGAbiolinks v2.35.0 [28]. Supplemental survival data were downloaded from the standardized dataset named the TCGA Pan-Cancer Clinical Data Resource (TCGA-CDR) [29]. We merged the survival data from TCGA-CDR with the GDC clinical data. We performed our tests on the overall survival (OS) endpoint. The corresponding TCGA-CDR columns included OS for status and OS.time for time-to-event data. The OS column contained the value 0, which encoded for alive (censored) status and 1 for deceased (failure), and OS.time contained the number of days from the date of diagnosis to either the date of last follow-up if OS was 0 or time to death if OS was 1.

We downloaded the fresh frozen (FF) RNA-seq data for the following TCGA projects of the “pan-gyn” group [25]: high-grade serous ovarian cystadenocarcinoma (TCGA-OV), uterine corpus endometrial carcinoma (TCGA-UCEC), cervical squamous cell carcinoma and endocervical adenocarcinoma (TCGA-CESC), uterine carcinosarcoma (TCGA-UCS), and invasive breast carcinoma (TCGA-BRCA). The harmonized GRCh38 aligned RNA-seq data (HTSeq-counts [30]) were normalized per TCGA project using the TCGAbiolinks normalization function, which is recommended for differential expression analysis. After merging RNA-seq and clinical data and discarding cases without survival information, we obtained 372 samples for OV, 1087 for BRCA, 549 for UCEC, 291 for CESC, and 55 for UCS. All the datasets shared 16,673 gene expression features.

#### 2.1.2. In-House Validation Dataset ICO-OV

We curated 12 patients diagnosed with high-grade serous ovarian carcinoma (HGSOC) between 2007 and 2016 at the Institut de Cancérologie de l’Ouest (ICO), and their retrospective electronic records data were collected: date of birth, date of pathologic diagnosis, clinical stage, histologic grade, date of death or date of last follow-up, the age at pathologic diagnosis. Based on these data the corresponding OS and OS.time variables were derived.

We extracted RNA from the corresponding archived formalin-fixed paraffin-embedded (FFPE) slides using the COVARIS ME220 Focused-ultrasonicator (COVARIS LLC, Woburn, USA) to ensure a high quantity of extracted RNA. RNA libraries were prepared using the SureSelect XT HS2 RNA Reagent kit and the SureSelectXT Human All Exon V6 + UTR probes from Agilent. All libraries were sequenced on an Illumina NextSeq 550 (Illumina Inc., San Diego, USA) in paired-end mode (2 × 75 bp) with a target depth of 20 million fragments per sample. Sequenced reads were trimmed with fastp v0.20.1 and mapped to GRCh38 using HISAT2 v2.1.0 both with default parameters. Reads overlapping genomic features were counted with featureCounts v2.0.0 [31] from the Subread package and Ensembl v99. Only uniquely mapped and not duplicated reads were counted. Multiple overlaps of unique genomic features were not counted.

The obtained raw featureCounts [31] of the ICO-OV dataset were further normalized using the TCGAbiolinks normalization function which resulted in 15521 genes in common between ICO-OV and the TCGA “pan-gyn” group. To account for the “batch effect”, we used the ComBat-seq method [32] from sva package v3.38.0, which is particularly suitable for RNA-seq data.

#### 2.1.3. External Validation Dataset JGOG-OV

In our study, we also used the JGOG-TR2 cohort [33,34], which was collected by the Japanese Gynecologic Oncology Group (JGOG). It consists of 274 high-grade serous ovarian carcinomas (HGSOCs) and 15 high-grade endometrioid carcinomas. The JGOG-TR2 FF RNA-seq data (GSE263455) are available on the NCBI GEO website (https://www.ncbi.nlm.nih.gov/geo/, accessed on 20 September, 2024), the clinical data are provided on the GitHub project page (https://github.com/shirotak/JGOG_HGEOC, accessed on 20 September, 2024), and the correspondence table between the RNA-seq and clinical identifiers were requested directly from the JGOG.

We normalized the GRCh38 aligned raw read counts of the JGOG-TR2 dataset using the DeSeq2 R/Bioconductor Package v1.44.0 [35] by taking into account the batch variable provided in the clinical data. The corresponding survival data were encoded to match the TCGA-OV and ICO-OV variables. For further analysis, we kept only HGSOC patients and the genes common between the ICO-OV and TCGA “pan-gyn” group, resulting in a dataset of 274 patients and 15518 genes (JGOG-OV dataset). To compare the clinical characteristics of the TCGA-OV, ICO-OV, and JGOG-OV datasets, we generated the descriptive statistics (see Table 1).

### 2.2. Proposed Deep Survival Model

In survival analysis, each data instance is described by a triplet $\left(X_{i},t_{i},d_{i}\right)$, where $X_{i}=\left(x_{i1},x_{i2},...,x_{ip}\right)$ is the feature vector for instance *i*, and $t_{i}$ is the observed time, time of failure if $d_{i}$ is 1 or right-censoring if $d_{i}$ is 0. For overall survival analysis (OS) with transcriptomic data, *X* is the gene expression matrix of *n* patients and *p* genes; if $d_{i}$ is 1, $t_{i}$ is the time from the initial diagnosis to death, and if $d_{i}$ is 0, $t_{i}$ is the time from the initial diagnosis to the last follow-up (censoring). The number of patients and genes in the different datasets is given in the previous Section 2.1.

Deep survival models are multi-layer artificial neural networks with different output layers that use various loss functions. The papers [14,15] give the overview of the different feed-forward survival models and their corresponding negative log-likelihood-based loss functions. The models Cox-nnet [7], SurvivalNet [16], VAECox [20], and SAVAE-Cox [23] all use the Cox negative partial log-likelihood as the loss function:

(1) $$\begin{array}{c} loss_{Cox}=\sum_{d_{j}=1}\left\{\theta_{j}\left(X_{j}\right)-log\left[\sum_{i\in R_{j}}exp\left(\theta_{i}\left(X_{i}\right)\right)\right]\right\} \end{array}$$

where $\theta_{j}\left(X_{j}\right)$ is the output of the neural network corresponding to the predicted risk of patient *j*, and $R_{j}$ is the set of indices *i*, with $t_{i}\geq t_{j}$ (patients at risk at time $t_{j}$). It is worth noting that in this loss function, the censored patients ($d_{i}=0$) do not contribute in the same way as the uncensored patients ($d_{i}=1$).

The discrete-time models are an alternative to the classical Cox method [15]. These models can be used as approximations of the models in continuous time by dividing time into *m* intervals. The probability mass function (PMF) method uses the following negative log-likelihood as a loss function that includes terms for the censored and the uncensored patient contributions:

(2) $$\begin{array}{c} loss_{PMF}=-\frac{1}{n}\sum_{i=1}^{n}\left(d_{i}log\left[\sigma_{k(t_{i})}\left(\phi \left(X_{i}\right)\right)\right]+\left(1-d_{i}\right)log\left[\hat{S}\left(k\left(t_{i}\right)|X_{i}\right)\right]\right), \end{array}$$

$$\mathrm{where}\;\sigma_{i}\left(\phi \left(X\right)\right)=-\frac{exp[\phi_{i}\left(X\right)]}{1+\sum_{k=1}^{m}exp[\left(\phi_{k}\left(X\right)\right]}\;\mathrm{is}\;\mathrm{the}\;\mathrm{softmax}\;\mathrm{function},\;$$

$$\phi \left(X\right)\;\mathrm{is}\;\mathrm{the}\;\mathrm{neural}\;\mathrm{network},\;k\left(t_{i}\right)\;\mathrm{is}\;\mathrm{the}\;\mathrm{interval}\;\mathrm{index}\;\mathrm{among}\;m\;\mathrm{intervals},$$

$$\mathrm{and}\;\hat{S}\left(k\left(t_{i}\right)|X\right)=1-\sum_{k=1}^{i}\sigma_{k}\left(\phi \left(X\right)\right)\;\mathrm{is}\;\mathrm{the}\;\mathrm{estimated}\;\mathrm{survival}\;\mathrm{function}.$$

Another discrete-time survival model DeepHit [11] uses the PMF loss function but combines it with a ranking loss. The composite loss function for one type of event can be written as

(3) $$\begin{array}{c} loss_{DeepHit}=\alpha loss_{PMF}+\left(1-\alpha \right)loss_{rank} \end{array}$$

(4) $$\begin{array}{c} loss_{rank}=\sum_{i,j}d_{i}1\left(t_{i}<t_{j}\right)exp\left(\frac{\hat{S}\left(k\left(t_{i}\right)|X_{i}\right)-\hat{S}\left(k\left(t_{i}\right)|X_{j}\right)}{\beta }\right), \end{array}$$

whereαandβarethehyperparametersofthenetwork.

Finally, the Neural Multi-Task Logistic Regression (N-MTLR) model [12] was shown by [14] to be equivalent to the PMF method in (2), but where

$$\phi_{j}\left(X\right)=\sum_{k=j}^{m}\psi \left(X_{k}\right)\;\mathrm{is}\;\mathrm{the}\;\left(\mathrm{reverse}\right)\;\mathrm{cumulative}\;\mathrm{sum}\;\mathrm{of}\;\mathrm{the}\;\mathrm{output}\;\mathrm{of}\;\mathrm{the}\;\mathrm{network}\;\psi \left(X_{k}\right).$$

We recently reported in [26] that the performance of the N-MTLR model was better than that of PMF [15] and Cox-nnet [7] for TCGA-OV transcriptome-based survival prediction. Therefore, we propose to test a variant of N-MTLR and call it N-MTLR-Rank. It combines the discrete-time negative log-likelihood of N-MTLR and the ranking loss of DeepHit:

(5) $$\begin{array}{c} loss_{N-MTLR-Rank}=\alpha loss_{N-MTLR}+\left(1-\alpha \right)loss_{rank} \end{array}$$

**Figure 1:**
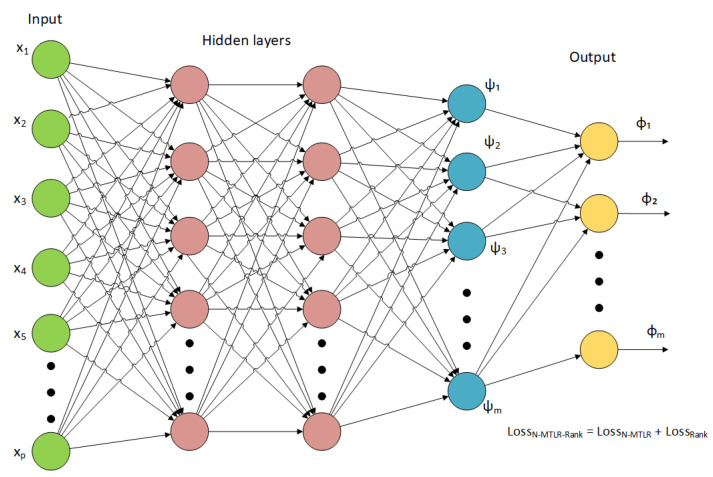
The N-MTLR-Rank model architecture. The input $X=(x_{1},x_{2},...,x_{p})$ of the network is the gene expression matrix, and the output ϕ(X) of the network serves to estimate the survival probability mass function for *n* time intervals. The particularity of the network is that the last layer is not fully connected, and instead, the reverse cumulative sum of ψ(X) is implemented as in the N-MTLR model. Plus, the loss function is composed of two parts, N-MTLR loss and ranking loss, similar to the DeepHit model.

Therefore, we train the feed-forward fully connected neural network as shown in Figure 1 and evaluate the composite loss function $loss_{N-MTLR-Rank}$ in Equation (5) as follows: first, we compute the reverse cumulative sum ϕ(X) of the network output ψ(X). We then estimate the survival function S(X). Finally, we compute the loss terms $loss_{N-MTLR}$ and $loss_{rank}$ using Equation (2) and Equation (4), respectively.

### 2.3. Model Training and Validation

We followed the same preprocessing steps as in our previous work [24,26] for RNA-seq data normalization. We applied the (log2+1) transformation to the normalized values, which is standard for gene expression data. For our tests, we only divided the TCGA-OV dataset into 5 folds using the R package MTLR v0.2.1 [36], thus creating 5 different splits into training and test sets with respectively 80% and 20% of the samples for a further 5-fold cross-validation. The split was performed using the stratification by the observed OS time (OS.time variable) and OS censoring (OS variable) to obtain similar distributions of survival times and censoring in training and test sets. To facilitate the training procedure, the training data were standardized to zero-mean and unit-variance to comply with the best practices for training deep learning algorithms. Note that in order to benefit from the multi-cancer transfer learning strategy, all the training in the following included BRCA+CESC+UCEC+UCS along with TCGA-OV samples.

For each of the five 80% TCGA-OV training sets, we performed the best hyperparameter combination search based on the Bayesian optimization technique as previously reported in [24]. We further divided the 80% training set into 60% optimization and 20% validation with the aim of training the networks with the optimization set and evaluating on the validation set. We used the Python library hyperopt v1.0.3 [27] for Bayesian optimization with the Adaptive Tree of Parzen Estimators (TPE) algorithm, a maximum number of trials of 400, and the following search space:

- Number of layers: 1–8.
- Layer width: 8–2048.
- Learning rate for Adam optimizer [37]: 0.00001–0.1.
- Weight decay [38]: 0–0.9.
- Dropout rate [39]: 0–0.6.
- Activation function: ReLU [40], SELU [41], hyperbolic tangent (tanh), sigmoid.
- Discretization scheme: equidistant or Kaplan–Meier quantiles [15].
- Interpolation scheme: constant density interpolation (CDI) or constant hazard interpolation (CHI) [15].
- α, ranking loss parameter in (4): 0–1.
- β, ranking loss parameter in (4): 0.1–100.

For our experiments, we used the DeepHit implementation of the Python package pycox v0.2.2 [15]. For N-MTLR-Rank, we computed the loss terms of Equation (5) using the N-MTLR implementation and the DeepHit ranking loss implementation of the same Python package.

The following hyperparameters were found for the best model: 1 hidden layer with 1131 nodes, learning rate of 0.00016, weight decay of 0.4678, dropout rate of 0.39, ReLU activation function, Kaplan–Meier quantiles discretization scheme, CDI interpolation scheme, α value of 0.25, and β value of 0.48. The best design of the network hyperparameters was then used to re-train a deep survival model using the 80% training set and the 20% test set to evaluate the concordance index (C-index) and Integrated Brier Score (IBS). We repeated this training procedure 10 times for each of the 5 train/test splits, thus resulting in the 50 trained and evaluated models. We reported the time-dependent C-index, which estimates the probability that the observations *i* and *j* are concordant, given that they are comparable. The C-index value of 0.5 is equivalent to a random guess, and 1 is the perfect concordance. As for the IBS, it is an extension of the Brier Score (BS) over a time interval, where BS is the mean squared error of the probability estimates. For this metric, smaller values indicate better performance; see [15] for more details.

### 2.4. Model Selection and Interpretation

The model used for interpretation was built by identifying the best-performing model configuration for the TCGA-OV and ICO-OV experiments. This configuration was then used to re-train a model using all available TCGA-OV samples. Feature attributions were calculated using the PatternAttribution method [42] implemented in PyTorch and available at https://github.com/KnurpsBram/PyTorch-PatternNet, accessed on 25 November 2021).

Feature attributions were analyzed using the R/Bioconductor package clusterProfiler v4.12.6 [43] for the Gene-Set Enrichment Analysis (GSEA) using the Molecular Signatures Database (MSigDB) [44] Hallmark collection (H) gene-sets.

The most abundant Hallmark gene-sets were aggregated into a combined 6 Hallmark pathways gene-set for Gene-Set Variation Analysis (GSVA). We used the GSVA function of the R/Bioconductor package GSVA v1.52.3 [45] and quantified the combined 6 Hallmark pathways’ activity in the RNA-seq data from the TCGA-OV, ICO-OV, and JGOG-OV datasets.

## 3. Results

### 3.1. Training and Comparing Deep Survival Networks

**Figure 2:**
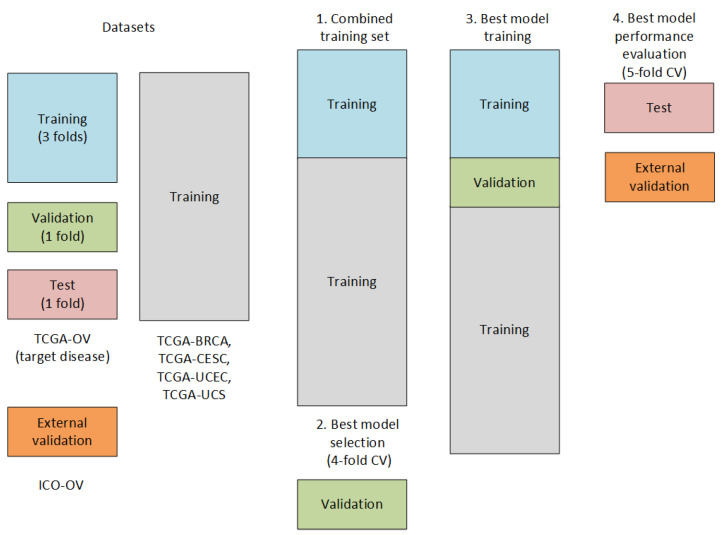
Transfer learning strategy. The target disease being ovarian cancer, the validation and test datasets from the TCGA-OV split serve to select the best model hyperparameters and evaluate the performance. The combined training dataset is composed of the training TCGA-OV and the four other “pan-gyn” datasets: TCGA-BRCA, TCGA-CESC, TCGA-UCEC, and TCGA-UCS. The external validation is performed on the in-house validation ICO-OV dataset.

In our previous experiments [24], we were able to demonstrate the ability of deep survival models to benefit from training on data from multiple cancer types. In this work, the survival models were trained using all five TCGA datasets of the “pan-gyn” group [25]: high-grade serous ovarian cystadenocarcinoma (TCGA-OV), uterine corpus endometrial carcinoma (TCGA-UCEC), cervical squamous cell carcinoma and endocervical adenocarcinoma (TCGA-CESC), uterine carcinosarcoma (TCGA-UCS), and invasive breast carcinoma (TCGA-BRCA). Survival networks were evaluated for their accuracy in predicting TCGA-OV outcomes, and the transfer learning strategy used is shown in Figure 2.

The deep survival models use the negative log-likelihood to adjust the weights of the neural network to transform molecular features into lower-dimensional latent variables to explain survival. Our recent experiments [26] showed the promising results of the survival model N-MTLR (Neural Multi-Task Logistic Regression) [12] in predicting the survival with ovarian high-dimensional gene expression profiles. In this paper, we tested the variant of the N-MTLR model, N-MTLR-Rank, in which we added the ranking loss to the negative log-likelihood of the N-MTLR model. The schema of the N-MTLR-Rank model is shown in Figure 2.

**Figure 3:**
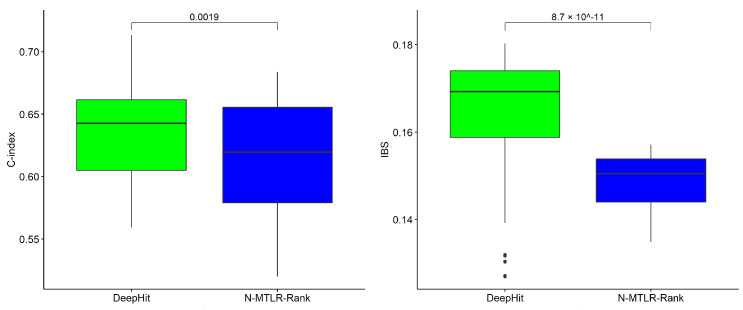
DeepHit and N-MTLR-Rank deep learning survival models’ performance comparison on the TCGA-OV dataset. The boxplots of the obtained 5-fold cross validation C-index (**left**) and IBS (**right**) on the test TCGA-OV datasets. The horizontal bars in the boxes represent the median values, the boundaries of the boxes delimit lower and upper quartiles, and the values outside the boxes are the lowest and the highest observations. Higher C-index values and smaller IBS values indicate better performance.

We compared the performance of our proposed method and the survival network DeepHit [11], which also uses the composite loss function. The obtained results (shown in Figure 3) show that N-MTLR-Rank significantly improves the Integrated Brier Score (IBS) (*t*-test *p*-value = $8.7\times 10^{-11}$) compared to DeepHit, while showing a worse but less significant performance in terms of the concordance index (C-index) (*t*-test *p*-value = 0.0019). Thus, our model overcomes the survival estimates calibration problem of DeepHit, which was pointed out by [14]. Indeed, DeepHit provides good performance in terms of C-index but at the cost of poorly calibrated survival estimates.

### 3.2. Validating with ICO-OV Dataset

**Figure 4:**
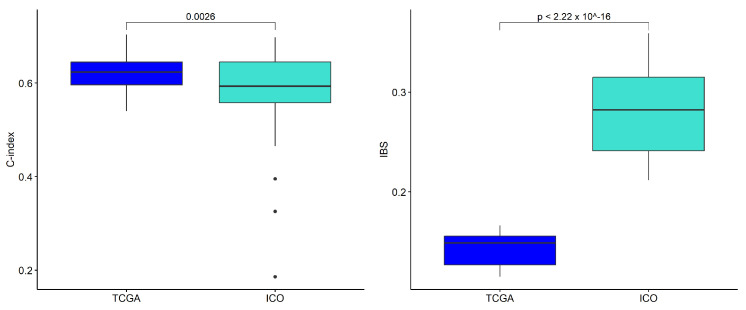
Evaluation of the generalization behavior of the N-MTLR-Rank model. The boxplots of the obtained 5-fold cross validation C-index (**left**) and IBS (**right**) on the test TCGA-OV and ICO-OV datasets, respectively. The horizontal bars in the boxes represent the median values, the boundaries of the boxes delimit lower and upper quartiles, and the values outside the boxes are the lowest and the highest observations. Higher C-index values and smaller IBS values indicate better performance.

We further evaluated the prognostic accuracy of the N-MTLR-Rank model on an in-house formalin-fixed paraffin-embedded (FFPE) dataset. For this purpose, we selected twelve high-grade serous ovarian carcinoma (HGSOC) patients from the Institut de Cancérologie de l’Ouest (ICO); we refer to this dataset as ICO-OV. The results of this generalization ability estimation are shown in Figure 4. We observed that the model N-MTLR-Rank does not generalize well to the new unseen data, when the resulting C-index performance may still be acceptable on the ICO-OV dataset (*t*-test *p*-value = 0.0026). This is clearly not the case for the IBS metric (*t*-test *p*-value < $2.22\times 10^{-16}$). Thus, even though the N-MTLR deep survival model outperforms the state-of-the-art Cox-nnet [26] and the N-MTLR-Rank model improves the performance of the DeepHit [11] on the TCGA-OV dataset, it cannot be directly used for survival prediction. Nevertheless, it is still important to investigate the features that the deep learning trained model uses for TCGA-OV prognostication, as they may serve to stratify ovarian cancer patients and provide more insights into the molecular mechanisms associated with survival.

### 3.3. Interpreting N-MTLR-Rank with PatternAttribution

The machine learning methods apply complex transformations to the input features, making the interpretation of these models difficult. Among the machine learning methods, the deep neural networks in particular are considered “black-boxes” because the input features in them undergo numerous nonlinear transformations. In order to provide a clear interpretation of the prognostic significance of individual features, we used the novel method called PatternAttribution [42]. The authors of this method demonstrated that the direction of the model gradients does not necessarily provide an estimate for the signal in the data. Instead, it reflects the relationship between the signal direction and the distracting noise contributions. They proposed a new decomposition method, PatternAttribution, by taking into account the data distribution. The measure of how much the input features contribute to the output through the layers in this method is called attribution. We generated the attributions for all input gene expressions for each patient in the TCGA-OV dataset in order to measure how the input features contribute to different outputs of the best trained network, i.e., survival probability mass function predictions.

To investigate the molecular pathways related to the ovarian cancer prognosis, we first performed the Gene-Set Enrichment Analysis (GSEA) [46] using the Molecular Signatures Database (MSigDB) [44]. The GSEA focuses on the coordinated differential expression of annotated groups of genes, or gene-sets, and provides results that are easier to interpret in terms of the relevant biological processes. We analyzed the attributions obtained for the OS endpoints close to 1 year (372 days) and to 5 years (1919 days) and ran the GSEA for all the patients in the TCGA-OV dataset. These attributions allowed the construction of the ranked lists of genes per patient, and the GSEA resulted in the list of significantly enriched Hallmark pathways per patient. The Hallmark collection (H) used for GSEA contains 50 gene-sets; we found 6 and 12 significantly enriched Hallmark pathways (Benjamini–Hochberg corrected *p*-value < 0.05). The percentage of the corresponding TCGA-OV patients is shown in Table 2.

Finally, we evaluated whether these molecular pathways could be used to identify high-risk or low-risk ovarian cancer patients. We selected the 6 most contributing Hallmark pathways (detected in more than 5% patients) at the 5-year endpoint: ALLOGRAFT REJECTION, EG2F TARGETS, ESTROGEN RESPONSE EARLY, G2M CHECKPOINT, IL2 STAT5 SIGNALING and MTORC1 SIGNALLING. Then, we performed the Gene-Set Variation Analysis (GSVA) [45]. The GSVA provides an estimate of the pathway activity by transforming the input gene-by-sample gene expression data into corresponding gene-set-by-sample expression data. We used the combined 6 Hallmark pathways gene-set and calculated its activity score per TCGA-OV patient. We estimated that approximately 30% of the TCGA-OV patients are alive at the 5-year stage, so we divided the patients into 30% low-risk and 70% high-risk groups based on the 6 Hallmark pathways’ activity score (the obtained cut-off threshold = 0.127). This TCGA-OV patient stratification resulted in a significant survival difference between the low- and high-risk groups (log-rank test *p*-value = 0.025).

**Figure 5:**
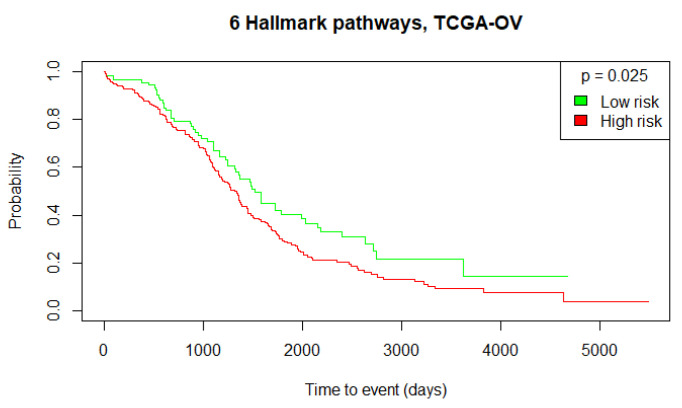
Kaplan–Meier curves of the high-risk and low-risk TCGA-OV patients. The groups were defined by calculating the 30% cut-off threshold of the 6 Hallmark pathways’ activity score of the TCGA-OV patients. The log-rank test was used to determine the statistical significance of the survival distributions between high-risk and low-risk groups (*p*-value ≤ 0.05).

**Figure 6:**
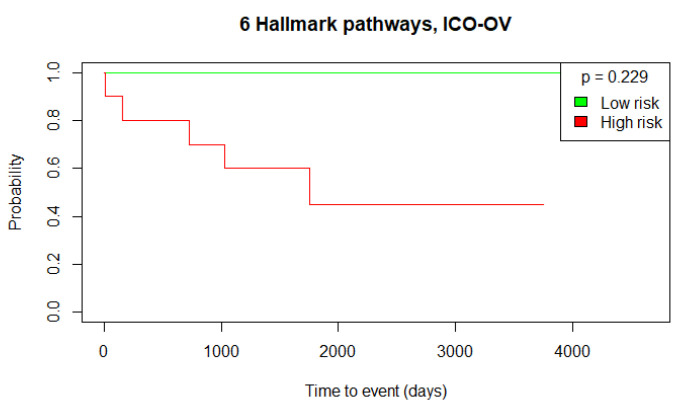
Kaplan–Meier curves of the high-risk and low-risk ICO-OV patients. The groups were defined by the cut-off threshold of the 6 Hallmark pathways’ activity score of the TCGA-OV patients. The log-rank test was used to determine the statistical significance of the survival distributions between high-risk and low-risk groups (*p*-value < 0.05).

**Figure 7:**
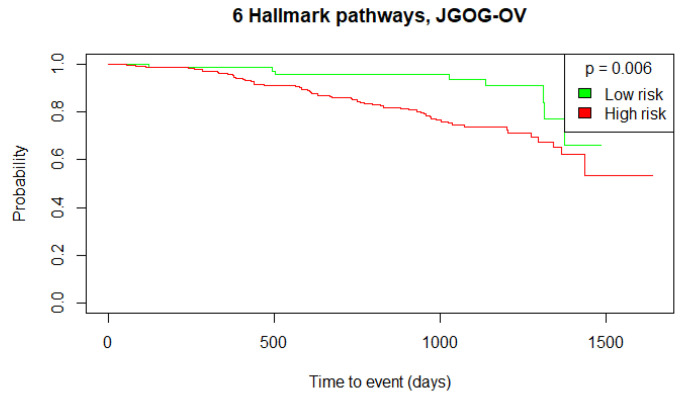
Kaplan–Meier curves of the high-risk and low-risk JGOG-OV patients. The groups were defined by the cut-off threshold of the 6 Hallmark pathways’ activity score of the TCGA-OV patients. The log-rank test was used to determine the statistical significance of the survival distributions between high-risk and low-risk groups (*p*-value < 0.05).

To further validate this stratification approach and the prognostic value of the identified Hallmark pathways, we performed the same GSVA using the independent validation datasets, ICO-OV and JGOG-OV. Using the TCGA-OV cut-off threshold, we obtained 2 low-risk versus 10 high-risk patients with no significant OS difference (log-rank test *p*-value = 0.229) for the ICO-OV dataset and 68 low-risk versus 206 high-risk patients with a significant OS difference (log-rank test *p*-value = 0.006) for the JGOG-OV dataset. The generated Kaplan–Meier curves are shown in Figure 5, Figure 6 and Figure 7. Given the Kaplan–Meier curve for the ICO-OV dataset, we hypothesize that the lack of significance may be due to the small size of the dataset.

## 4. Discussion

We propose a new variant of the deep survival model and evaluate its ability to learn from the high-dimensional transcriptomic profiles to predict the clinical outcomes. The N-MTLR-Rank model overcomes the time-invariant covariate effect requirement of the Cox Proportional Hazards models [14], providing the survival estimates for multiple time endpoints. It also uses the censored and uncensored data during the training to adjust the neural network weights, which helps to overcome the uncensored proportion drawback of the Cox-based models [7,16].

We argue that its predicting accuracy performance is due to the fact that its probability estimate at time *t* is a function of the probability estimates at times *t’>t*. Interestingly, it is the opposite of the RNN-SURV method proposed by [47] and based on the Long Short-Term Memory (LSTM) [48] cells, which exploit the sequential nature of the problem, but nevertheless N-MTLR-Rank still performs well. Indeed, the N-MTLR-Rank model resulted in significant improvements in terms of IBS compared to the DeepHit model while resulting in worse but less significant performance in terms of the C-index.

We evaluate the generalization capabilities of the N-MTLR-Rank deep learning survival model on the new unseen data, coming from RNA-sequencing of the archived formalin-fixed paraffin-embedded (FFPE) samples, which could open the possibility of using other retrospective cohorts. We observe that while the resulting C-index performance on the in-house FFPE ICO-OV dataset may still be acceptable, it is not the case for the IBS metric. We conclude that the trained N-MTLR-Rank model may not be directly used for survival prediction.

We also experiment with new methods for model interpretation, such as PatternAttribution [42]. This methodology allows us calculate the gene expression attributions used by the best trained neural network model to predict outcomes. We perform the GSEA analysis using these input feature attributions and report the most frequently enriched pathways of the MSigDB hallmark (H) collection. We analyze the six significantly enriched pathways that are most present in TCGA-OV patients: ALLOGRAFT REJECTION, EG2F TARGETS, ESTROGEN RESPONSE EARLY, G2M CHECKPOINT, IL2 STAT5 SIGNALING, and MTORC1 SIGNALLING.

Among these pathways, IL2 STAT5 SIGNALING, ESTROGEN RESPONSE EARLY, G2M CHECKPOINT, and MTORC1 SIGNALING have already been reported to be prognostically enriched by the authors of [16].

The found immune activation pathways, such as ALLOGRAFT REJECTION and IL2 STAT5 SIGNALING, are strongly associated with better survival. This observation is consistent with the results of another study, where the authors clustered the TCGA-OV patients into immune subtypes [4] based on the manually curated immune-related genes and reported that these pathways had higher activation in immune subtype 1, which was associated with better survival.

The ESTROGEN RESPONSE EARLY pathway is a set of genes that regulate an early response to estrogen. Given that the estrogen receptor alpha (ER-alpha, *ESR1*) is known to be the major mediator of the estrogen response [49], we hypothesize that the TCGA-OV subpopulation that was detected to be enriched for this pathway in our survival model may provide a plausible basis for a future biomarker study of anti-estrogen therapies.

Our findings that the proliferation pathways G2M CHECKPOINT and E2F TARGETS are associated with survival are in line with the study [8], where the authors found these pathways to be significantly enriched in ovarian cancer, suggesting that they may play a critical role in the development of ovarian cancer. The previous findings in the literature reviewed by [8] suggest that the E2F family is crucial for cancer initiation, progression, and resistance to therapy. The signaling pathway MTORC1 SIGNALING is frequently activated in ovarian tumors and plays an important role in tumor metabolism [50] and in the differentiation and function of immune cells [51]. Therefore, the mTOR signaling pathway is a hot target in anti-tumor therapy research.

Finally, the activity of these 6 Hallmark pathways could help in the stratification of ovarian cancer. Indeed, the recent study of the long-term survivors of high-grade serous ovarian cancer [52] described a variety of factors involving the patient’s genome, tumor somatic mutational profile, and immune response. This finding is consistent with the diversity of Hallmark pathways found in our work and the fact that the combined activity of the Hallmark pathways results in the significant patient stratification in the external independent validation cohort.

Understanding and advancing the treatment of ovarian cancer is conditioned by the identification of the underlying biology and molecular pathogenesis of this disease. Although our study extends the insights into the use of deep learning for survival modeling and the found prognostic associated molecular pathways of ovarian cancer patients represent a promising point for future research, it is worth underlining that these pathways should be considered as hypothesis generators, and more detailed in vitro experiments and follow-up clinical studies are required.

## 5. Conclusions

Deep learning-based survival prediction of cancer patients using high-dimensional gene expression data has gained recognition in contemporary medical research. While these models show good performance on a holdout test dataset coming from the same distribution as the training dataset, they fail to generalize well to new out-of-distribution validation datasets. Nevertheless, even if the direct application of deep learning-based models for survival prediction with RNA-seq data is not yet foreseeable in the current clinical practice, these models could still be used as hypothesis generators, and the detection and interpretation of the gene-sets that contribute the most to the survival output predicted by these models open a new research direction. The detected molecular pathways, which have been shown to be biologically relevant, could be used for patient stratification, and the external validation of fresh frozen and formalin-fixed paraffin-embedded RNA-seq datasets can further confirm their stratification ability. 

## Figures and Tables

**Table 1:** TCGA- OV, ICO-OV, and JGOG-OV clinical descriptive statistics.

Variable	TCGA-OV	ICO-OV	JGOG-OV
Age at pathologic diagnosis			
Count	372	12	274
Mean (SD)	59.60 (11.38)	63.17 (12.64)	61.22 (11.41)
Median (IQR)	59.00 (17.00)	67.50 (20.50)	61.50 (15.00)
Q1, Q3	51.00, 68	50.50, 71	54.00, 69
Min, Max	30.00, 87	46.00, 86	28.00, 89
Missing	0	0	0
Clinical stage			
Count (%)	372	12	274
Stage IB		1 (8.33%)	
Stage IC	1 (0.27%)		
Stage II		1 (8.33%)	
Stage IIA	3 (0.81%)		7 (2.55%)
Stage IIB	3 (0.81%)		19 (6.93%)
Stage IIC	15 (4.03%)		
Stage IIIA	7 (1.88%)	2 (16.67%)	19 (6.93%)
Stage IIIB	13 (3.49%)		32 (11.68%)
Stage IIIC	270 (72.58%)	7 (58.33%)	138 (50.36%)
Stage IV	57 (15.32%)	1 (8.33%)	53 (19.34%)
Missing	3 (0.81%)	0	6 (2.19%)
**Histological grade**			
Count (%)	372	12	274
G1	1 (0.27%)		
G2	42 (11.29%)		
G3	319 (85.75%)	12 (100%)	274 (100%)
G4	1 (0.27%)		
GB	2 (0.54%)		
GX	5 (1.34%)		
Missing	2 (0.54%)	0	0
**OS**			
Count (%)	372	12	274
0	143 (38.44%)	7 (58.33%)	210 (76.64%)
1	229 (61.56%)	5 (41.67%)	64 (23.36%)
Missing	0	0	0
**OS.time**			
Count	372	12	274
Mean (SD)	1187.17 (943.74)	2132.75 (1609.92)	1021.62 (321.42)
Median (IQR)	1024.00 (1141.75)	1677.50 (2786.50)	1072.00 (297.00)
Q1, Q3	517.25, 1659	954.00, 3740.5	932.25, 1229.25
Min, Max	8.00, 5481	8.00, 4646	56.00, 1639
Missing	0	0	0

**Table 2:** The MSigDB Hallmark pathways found to be significantly enriched based on the TCGA-OV genes attributions (*p*-value < 0.05). The attributions were obtained using the best-performing deep survival network N-MTLR-Rank trained with TCGA-OV data. The 6 pathways detected in more than 5% of TCGA-OV patients are shown in bold.

Collection	TCGA-OV, 1 y.	TCGA-OV, 5 y.
HALLMARK_ALLOGRAFT_REJECTION	0.54%	**25.81%**
HALLMARK_E2F_TARGETS	-	**13.98%**
HALLMARK_ESTROGEN_RESPONSE_EARLY	0.81%	**6.72%**
HALLMARK_ESTROGEN_RESPONSE_LATE	-	2.42%
HALLMARK_G2M_CHECKPOINT	-	**8.33%**
HALLMARK_HYPOXIA	0.27%	1.88%
HALLMARK_IL2_STAT5_SIGNALING	2.96%	**20.97%**
HALLMARK_MTORC1_SIGNALING	0.54%	**5.91%**
HALLMARK_MYC_TARGETS_V1	-	3.23%
HALLMARK_PANCREAS_BETA_CELLS	1.34%	1.61%
HALLMARK_UNFOLDED_PROTEIN_RESPONSE	-	0.54%
HALLMARK_WNT_BETA_CATENIN_SIGNALING	-	0.27%

## Data Availability

The TCGA (The Cancer Genome Atlas) datasets are available using the following link: https://portal.gdc.cancer.gov/, accessed on 19 February 2021. All data not published in the tables and supplements of this article are available from the corresponding author on request.

## Abbreviations

The following abbreviations are used in this manuscript:

BRCA	Breast cancer gene or Invasive Breast Carcinoma
BS	Brier Score
CDI	Constant Density Interpolation
CESC	Cervical Squamous Cell Carcinoma and Endocervical Adenocarcinoma
CHI	Constant Hazard Interpolation
C-index	Concordance Index
FFPE	Formalin-Fixed Paraffin-Embedded
FF	Fresh Frozen
GDC	Genomics Data Commons
GSEA	Gene-Set Enrichment Analysis
GSVA	Gene-Set Variation Analysis
HGSOC	High-Grade Serous Ovarian Carcinoma
HRD	Homologous Recombination Deficiency
IBS	Integrated Brier Score
ICO	Institut de Cancérologie de l’Ouest
LSTM	Long Short-Term Memory
MSigDB	Molecular Signatures Database
MTLR	Multi-Task Logistic Regression
N-MTLR	Neural Multi-Task Logistic Regression
OV	High-Grade Serous Ovarian Cystadenocarcinoma
PARP	Poly(ADP-Ribose) Polymerase
PMF	Probability Mass Function
TCGA	The Cancer Genome Atlas
TCGA-CDR	TCGA Pan-Cancer Clinical Data Resource
UCEC	Uterine Corpus Endometrial Carcinoma
UCS	Uterine Carcinosarcoma
